# Rest as an Important Factor in the Nonoperative Treatment of Gynecological Disorders1Read before the Allegheny County Medical Society, October 15, 1907.

**Published:** 1908-10

**Authors:** Edward A. Weiss

**Affiliations:** Pittsburg, Pa.


### Rest as an Important Factor in the Nonoperative Treatment of Gynecological Disorders.[1]

By EDWARD A. WEISS, M. D., Pittsburg, Pa.

(*Pennsylvania Medical Journal*, September, 1908.)

THE advances made in modern gynecology have been so distinctly surgical that many nonoperative procedures have been neglected. This is clearly exemplified in many of the recent textbooks which devote little or no space to the palliative treatment of pelvic disorders. The surgical treatment alone is taught so that many valuable therapeutic means have undeservedly fallen

1. Read betore the Allegheny County Medical Society, October 15, 1907.

into disuse. The result is that too often needless and sometimes harmful operations are performed on the female pelvic organs. By such unnecessary operations not only are the patients not relieved but the profession is criticised, and often justly so.

There are many pelvic conditions which yield only to surgical treatment, and in which therapeutic and other palliative measures would be useless and sometimes harmful; as, for instance, in growing neoplasms, pus accumulations or complicated displacements. In other cases the patient may choose between a long continued nonoperative or an operative plan of treatment. A third class of cases embraces medical as well as operative treatment in order to secure the best results. In still other cases non-operative measures only are necessary.

Before deciding on any line of treatment for the correction of pelvic disorders, a most careful and painstaking examination must be made. The best results are to be had not always by the one who operates most, but by the one who knows when not to operate. Certain operations easy of execution are often employed as a routine measure when nonoperative means would be productive of more good. Possibly of all operations none is more frequently abused than the operation of curetment. Not infrequently a simple functional derangement is converted into a real pathological condition by the indiscriminate use of the curet.

The uselessness of drugs in many pelvic disturbances is well known. That no drug can have an elective action on the tubes, ovaries or uterine mucosa is well known. Yet in spite of this knowledge many standard as well as proprietary drugs are prescribed for endometritis, salpingitis or inflammatory conditions of the pelvis. But the fact must always be borne in mind that certain remedies by their general systemic action influence certain local pelvic conditions; as, for example, a brisk purge may by its depleting action often relieve pelvic congestion and painful menstruation, or the proper employment of iron and arsenic may be the very best remedy for the correction of certain functional menstrual disorders.

Some of the more generally adopted measures in the palliative treatment of gynecological disorders in addition to medication are elimination, douches, local applications, tampons, massage and electricity. But the most important general agent in the treatment of female disorders is rest. This is a factor which has not been given the attention it deserves.

In this connection rest not only implies cessation from work but also from functional activity. When we tell the average patient that she should rest it is interpreted to mean lounging about the house with no definite routine of living. When we prescribe rest we should explain minutely what the patient should do, just as we describe minutely the diet of a dyspeptic.

Rest is required in a number of conditions in gynecology but the more common instances are (1) in acute inflammatory conditions; (2) in congestive pelvic disorders; (3) in certain displacements complicated by splanchnoptosis; (4) in nervous disorders referable to the pelvis, especially neurasthenia.

According to the indications rest may be (1) absolute, continuous rest in bed, (2) partial rest in bed, (3) rest at stated intervals, (4) rest with exercise, or (5) isolation.

The principles of rest were first pointed out by Hilton in his lectures on Rest and Pain in 1860. He stated that "it is an admitted physiological axiom that each structure or organ, whilst actually employed, is in a state of vascular excitement or turgescence and, therefore, enlarged during that time. So it is noticeable that each organ of the body which is liable to the rapid supervention of activity in its proper function is so placed in its relation to surrounding structures as to permit of temporary enlargement during the persistence of that activity. When it returns to its state of rest or period of self-reparation, it may be said to have returned to its normal or standard dimensions."

We know from clinical experience that rest in bed decreases the amount of blood sent to the part, and also lessens the force of circulation. It has been demonstrated that the heart beats about fifteen times a minute less when the patient is recumbent than when he is erect. The benefit to the local condition and the consequent relief from congestion is, therefore, great.

Rest in connection with acute pelvic infection should be absolute. The patient should not be allowed to leave her bed under any circumstances; neither should she be allowed to sit up in bed. The necessity of such absolute rest has been proved repeatedly in cases of acute pelvic peritonitis which were treated at the patients' homes, for three or four weeks without the temperature remaining normal longer than a few days at a time. The sending of the patient to the hospital or the placing of a competent nurse in charge of the patient would repeatedly result in the temperature remaining normal, with a continued improvement in the inflammatory condition. The improvement in every case could be traced to the fact that the patient was kept at absolute rest under direct supervision. The patient, unless instructed to the contrary, does not consider sitting up in bed or getting up to empty the bladder or bowels detrimental to speedy recovery. In treating pelvic inflammatory conditions, other measures should of course be employed, such as elimination, ice to the abdomen, diet, etc., but rest is of imperative value.

Our present knowledge of the acute pelvic infections is such that much local treatment, particularly by operative interference, should be employed only in a small percentage of cases during the acute stage of the affection. Formerly we were taught that the presence of pus was an indication for immediate operation. We now know from clinical experience that in such cases, when treated by absolute rest, the infection becomes less virulent and that the bacteria and toxins disappear. Unless secondary infection occurs, sepsis usually disappears in from two to three weeks.

Until about four years ago it was the rule to operate at almost any stage of pelvic infection. A comparison of our postoperative mortality as well as morbidity with the present method of waiting two or three weeks after all evidence of sepsis has disappeared, shows a marked improvement in favor of the later method of treatment.

The necessity of rest in cases of bleeding is well known, nevertheless it is a therapeutic measure which is too often neglected in the treatment of menorrhagia, subinvolution or other uterine bleedings. Ergot, hydrastis and other similar drugs are notoriously inefficient when not supplemented by rest. In like manner, pelvic engorgement with its accompanying pelvic pain is best relieved by absolute rest. If such a plan of treatment were instituted in selected cases of so-called congestive endometritis, many needless curetments would be avoided.

Posterior displacements and partial or aggravated prolapsus of the uterus, especially when associated with ptosis of the abdominal viscera, are undoubtedly relieved, but of course not cured, by the recumbent position. While more of such cases should be treated by surgical measures only, there are conditions such as age, constitutional disease or even family considerations which do not allow of operative treatment. In such patients it would be an injustice to disregard the case by denying that there is any other relief than by the knife. If the case is one of retroposition of the uterus without any complication, the treatment should be reposition of the uterus together with a properly fitting pessary. To prevent undue pressure and at the same time to avoid the pelvic venous stasis, which attends such conditions, rest in the recumbent position for at least one hour every afternoon should be insisted upon as a most important adjunct to the treatment. This form of treatment is particularly applicable to the all too numerous class of patients who for social and financial reasons must work without sufficient relaxation. Such patients feel relieved when lying down and for a few hours afterward, but standing constantly for six or eight hours causes a return of the symptoms and if the standing position is maintained for ten or twelve hours the condition is more aggravated and the distress is consequently greater.

Pelvic disorders and neurasthenia are so often closely associated that frequently it is almost impossible to determine which is cause and which is effect. Any diseased condition should of course be given the proper surgical and medical attention before pronouncing it neurasthenia. It is in this class of patients particularly that the note of warning should be sounded against operating for pelvic disorders with the expectation of relieving the patient of her neurasthenic symptoms. The treatment of pelvic neurasthenia presents a varied problem to the attending physician. It would be advisable in many cases to treat the patient for several days before operating, if such a procedure is indicated. In this way the symptoms can be studied closely and the rest would be of decided benefit in many ways. This treatment could best be conducted with the patient in bed, and better in a hospital where she will be away from home influence. In a few cases, a systematic Weir Mitchell rest treatment should be tried. If the patient responds to treatment there should be an increase of from two to four pounds a week. The benefit is often extraordinary not only as to the neurasthenia but also as to the pelvic condition.

The advantage of the rest treatment can best be demonstrated by the improvement shown by some patients during the three or four weeks in bed after operation. The rapid improvement is due not so much to the operation as to the enforced rest, for example, cases of extreme prolapsus uteri with its long train of symptoms, such as emaciation, gastric and intestinal distress, nervous derangement, and the like; and it would be no exaggeration to say that such a patient derives as much good from the prolonged rest in bed as from the operation itself.

The only objection to this long continued inaction is that certain objectionable effects would manifest themselves, such as poor elimination, inactivity of muscles, accumulation of fat, etc. The most important measure to overcome these defects is the employment while in bed of regular systematic massage or manipulation of the muscles, by which means a healthy condition can be maintained until such time when regular muscular exercise can be resumed.

In regard to bodily exercise the index should be the strength and general condition of the patient. A mistake too often made is to make weak and debilitated patients take long walks. For such a patient rest is more desirable and fresh air may be had at the same time.

It may seem from the foregoing that I am opposed to operative treatment in gynecological conditions, but such is not the case. Let us operate for true surgical conditions when a real indication exists.

The suggestions offered are not new, but the principle of rest is too often neglected or is relegated to a minor place in our treatment of gynecological patients, and the object of this paper is to emphasize a simple but most important adjunct to our nonoperative treatment of pelvic disorders.

DISCUSSION.

Dr. L. Litchfield, Pittsburg: It is very refreshing to hear the nonsurgical treatment of gynecological cases so exhaustively and suggestively discussed by a gynecologist. Dr. Weiss's most interesting paper shows us that at least one gynecologist appreciates fully the great value and importance of nonsurgical treatment in many of these cases. I am neither a gynecologist nor obstetrician, but I wish to bear witness to what Dr. Weiss has said regarding rest in these cases, and I wish particularly to call attention to the value of the knee-chest position combined with rest in many cases of chronic displacement, and, particularly, a postpartum case which showed a tendency to prolapse and retrodisplacements; also, as to the value of the pessary worn for a few months in some of these post-partum cases.

Dr. K. I. Sanes, Pittsburg: The importance of rest in acute pelvic inflammatory diseases has lately been recognised by the gynecological profession. Rest improves the general health of the patient and puts her in better condition for the operation. Having allayed the acute inflammation, it enables us during the operation to distinguish the diseased from the healthy tissues. It also enables us to study better our patient and her disease, and thus to correct our incomplete or faulty diagnosis. Even complete cure can at times be attributed to rest. But to get the benefit of the preliminary rest treatment and at the same time not to run any unnecessary risk of complications and accidents, it should be undertaken at the hospital under supervision of the surgeon and under care of a nurse so that sudden changes demanding immediate intervention may not be overlooked.

In this connection it is interesting to note the change that has been taking place lately in the postoperative treatment so far as rest is concerned. Instead of keeping our patients in bed three weeks after serious operations, we let them sit up on second or third day, let them walk on the fifth or sixth day and send them home at the end of the second week. Of course, we must use our judgment in this matter. Patients of low vitality, neurasthenics, hernias, and the like, must have their usual postoperative rest, but the severity of the operation itself bears hardly any relation to the length of time necessary for postoperative rest.

